# *Oroxylum indicum* ameliorates D-galactose-induced aging related memory impairments via enhancing rat hippocampal neurogenesis

**DOI:** 10.1038/s41598-025-27042-5

**Published:** 2025-12-02

**Authors:** Nittaya Tanrangka, Ram Prajit, Soraya Kaewngam, Tanaporn Anosri, Worapol Sae-Foo, Anusara Aranarochana, Nataya Sritawan, Apiwat Sirichoat, Waraporn Putalun, Peter Wigmore, Jariya Umka Welbat

**Affiliations:** 1 Department of Anatomy, Faculty of Medicine, Khon Kaen University, Khon Kaen, 40002 Thailand; 2 Faculty of Pharmaceutical Sciences, Khon Kaen University, Khon Kaen, 40002 Thailand; 3 Department of Pharmacognosy and Pharmaceutical Botany, Faculty of Pharmaceutical Sciences, Prince of Songkla University, Hat Yai, Songkhla, Thailand; 4 School of Life Sciences, Queen’s Medical Centre, Medical School, University of Nottingham, 13, Nottingham, NG7 2RD UK

**Keywords:** *Oroxylum indicium*, D-galactose, Brain aging, Memory, Hippocampal neurogenesis, Neuroscience, Senescence

## Abstract

Brain aging significantly affects human lives, particularly among the elderly, leading to various diseases and constituting memory and cognitive decline. Hippocampal neurogenesis, the process of generating new neurons throughout life, decline with aging, which contribute to cognitive decline and dementia. D-galactose (D-gal) induces brain aging by increasing oxidative stress and inflammation, resulting in neurodegeneration and memory dysfunction. *Oroxylum indicum* fruit extract (OIFE), a traditional Thai herb, possesses strong antioxidant and neuroprotective properties. We investigated whether OIFE extract mitigates D-gal-induced memory decline, assessing its phytochemical profile, antioxidant capacity and neuroprotective effects. The results revealed that OIFE possessed strong phytochemical properties using measures such as total phenolic content (TPC), total flavonoid content (TFC), 2,2-diphenyl-1-picrylhydrazyl (DPPH), ferric reducing antioxidant power (FRAP), and high-performance liquid chromatography–ultraviolet (HPLC–UV). Liver and kidney function tests on blood samples showed no significant differences among the groups. For memory function testing, sixty male Sprague Dawley rats were divided into six groups and treated with OIFE (125 or 250 mg/kg, p.o.) and D-gal (50 mg/kg, i.p.) for eight weeks. D-gal administration resulted in significant memory deficits (accessed via the novel object location [NOL] and novel object recognition [NOR] test). These deficits were correlated with a decrease in the number of immature neurons and reduced neuronal cell survival in the subgranular zone (SGZ), as observed using doublecortin (DCX) and 5-Bromo-2′-deoxyuridine/Neuronal nuclear protein (BrdU/NeuN) immunofluorescence staining, respectively. However, co-treatment with OIFE ameliorates D-gal-induced these impairments. Therefore, OIFE could mitigate memory and neurogenesis impairments in D-gal-induced brain aging in rats.

**Supplementary Information:**

The online version contains supplementary material available at 10.1038/s41598-025-27042-5.

## Introduction

The proportion of the elderly population, defined as people aged 60 and over, is expected to increase from 12.3% to 21.5% over the next few decades^1^. Aging is a process of cell senescence that affects organ functions, especially brain functions^2^. The aging process induces cell damage related to impairment of structures and functionals in the body. Brain aging is the main cause of neurodegenerative diseases, such as dementia, Alzheimer’s disease, and Parkinson’s disease^2,3^. In addition, memory and cognitive impairments are commonly found in aging populations, leading to a decrease in quality of life and increased weakness disability^4^. Neurodegeneration is often associated with abnormal homeostasis activities, for instance, DNA damage, protein, lipid, and mitochondrial dysfunction^3^. Previous studies have postulated that age-related decline in memory function is associated with decreased neurogenesis^5,6^. Neurogenesis is an essential system for generation of new neurons in the brain, particularly in the subgranular zone (SGZ) of the hippocampus. Normally, hippocampal neurogenesis involves neural stem cell (NSC) proliferation, transformation into immature neurons, and migration to the granule cell layer^5,7^. Decreasing hippocampal neurogenesis is influenced by neuroinflammation, oxidative stress, and lipid peroxidation^8,9^.

Chronic administration of D-galactose (D-gal) in animal models produces an aging-like phenotype in the brain by triggering brain cell deterioration, leading to memory impairment^10–13^. Chronic administration of D-gal-generated reactive oxygen species (ROS) causes oxidative stress and induces neuronal cell damage^14,15^. Accumulation of D-gal leads to the generation of advanced glycation end-products, which increase inflammation and oxidative damage in the brain. Additionally, long-term D-gal-induced cell deterioration involves unstable oxidant production and antioxidant enzyme activity, which contribute to acceleration of aging. D-gal generates ROS through galactose oxidase activity, leading to a progressive decline in learning and memory function. D-gal induces memory impairment, particularly spatial and recognition memory, by reducing neurogenesis and causing cell apoptosis in the brain^16–18^. Furthermore, administering D-gal at a dose of 50 mg/kg for 8 weeks can reduce both spatial and recognition memory, which are linked to hippocampal neurogenesis^15,19,20^. Although D-gal-induced brain aging, driven by oxidative stress, leads to impaired hippocampal neurogenesis, new therapeutic options may slow down or improve this process.

Natural products contain flavonoids that have a neuroprotective effect associated with neurogenesis in the brain aging. *Oroxylum indicum* (L.) Benth. ex Kurz (*O. indicum*), found in Asia, such as Thailand^21^, Sri Lanka, Philippines, Indonesia, China, and Malaysia, is referred to as Pheka in Thailand. All parts of *O. indicum* can be used in traditional medicine and as food ingredients^22,23^. It also exhibits pharmacological properties, including antioxidant and anti-inflammatory effects that help alleviate oxidative stress. *O. indicum* contains bioactive compounds that belong to flavonoid group, such as oroxylin A, baicalein, and chrysin^22–24^. A prior study has found that bark and leave extracts of *O. indicum* improve memory impairments, decrease ROS, and lipid peroxidation, and increase antioxidant levels caused by doxorubicin chemotherapy in a mice model^25,26^. Studies have investigated the neuroprotective effects and the mechanisms of *O. indicum* extracts on memory impairment in animal models of chemotherapy and cognitive function in older adults^26,27^. Therefore, this inquiry aims to investigate the phytochemical analysis, antioxidant activity, and the neuroprotective effect of *O. indicum* fruit extract against memory and hippocampal neurogenesis declines in the SGZ of the hippocampal dentate gyrus in D-gal-induced aging rats.

## Results

### Phytochemicals of the OIFE

**Fig. 1:**
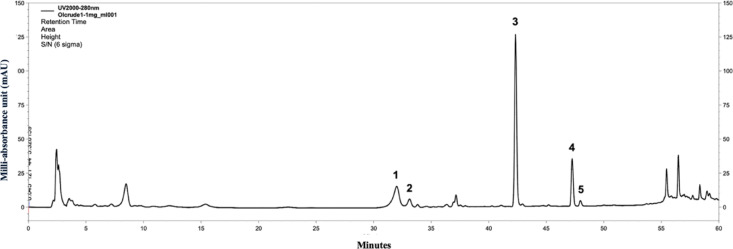
HPLC chromatogram of standardized OIFE. Peaks labeled with 1–5 represent the major compounds, including (1) oroxin A, (2) baicalin, (3) baicalein, (4) chrysin, and (5) oroxylin A.

The results demonstrated that OIFE contained a high level of TPC, measuring 73.506 ± 19.2 mg GAE/g extract powder and TFC, with a content of 125.16 ± 1.20 mg QE/g extract powder content. The DPPH IC_50_ values for L-ascorbic acid and OIFE were 3.10 ± 0.27 μg/ml and 122.18 ± 0.33 μg/ml, respectively. Moreover, the antioxidant activity in OIFE showed a high level of FRAP, with a content of 116.97 ± 0.002 (Fe^2+^ μg/g extract). The results of the phytochemical analysis of OIFE indicated that it possesses significant free radical scavenging and antioxidant activity. HPLC–UV analysis of OIFE, conducted using a modified version of a previously published method showed five major peaks in the extract as presented in Fig. 1^28^. The major compounds in OIFE include (1) oroxin A, (2) baicalin, (3) baicalein, (4) chrysin, and (5) oroxylin A. The identified compounds of OIFE data were presented in Table 1. The precision of HPLC–UV system was evaluated as 0.46–1.80 percentage relative standard deviation (%RSD) for intra-day precision and 0.67–2.94%RSD for inter-day precision as shown in Table S1. The precision was in acceptable criteria.

**Table 1:** Main compound of OIFE identified through HPLC–UV analysis.

Peak	Retention time (min)	Compounds	Contents (mg/g)
1	33.019 ± 0.984	oroxin A	22.01 ± 1.36
2	33.883 ± 0.749	baicalin	4.06 ± 0.32
3	42.522 ± 0.195	baicalein	28.67 ± 1.90
4	47.341 ± 0.098	chrysin	4.48 ± 0.37
5	48.041 ± 0.091	oroxylin A	0.69 ± 0.07

### Effect of OIFE on body weight, as well as kidney and liver function tests

**Fig. 2:**
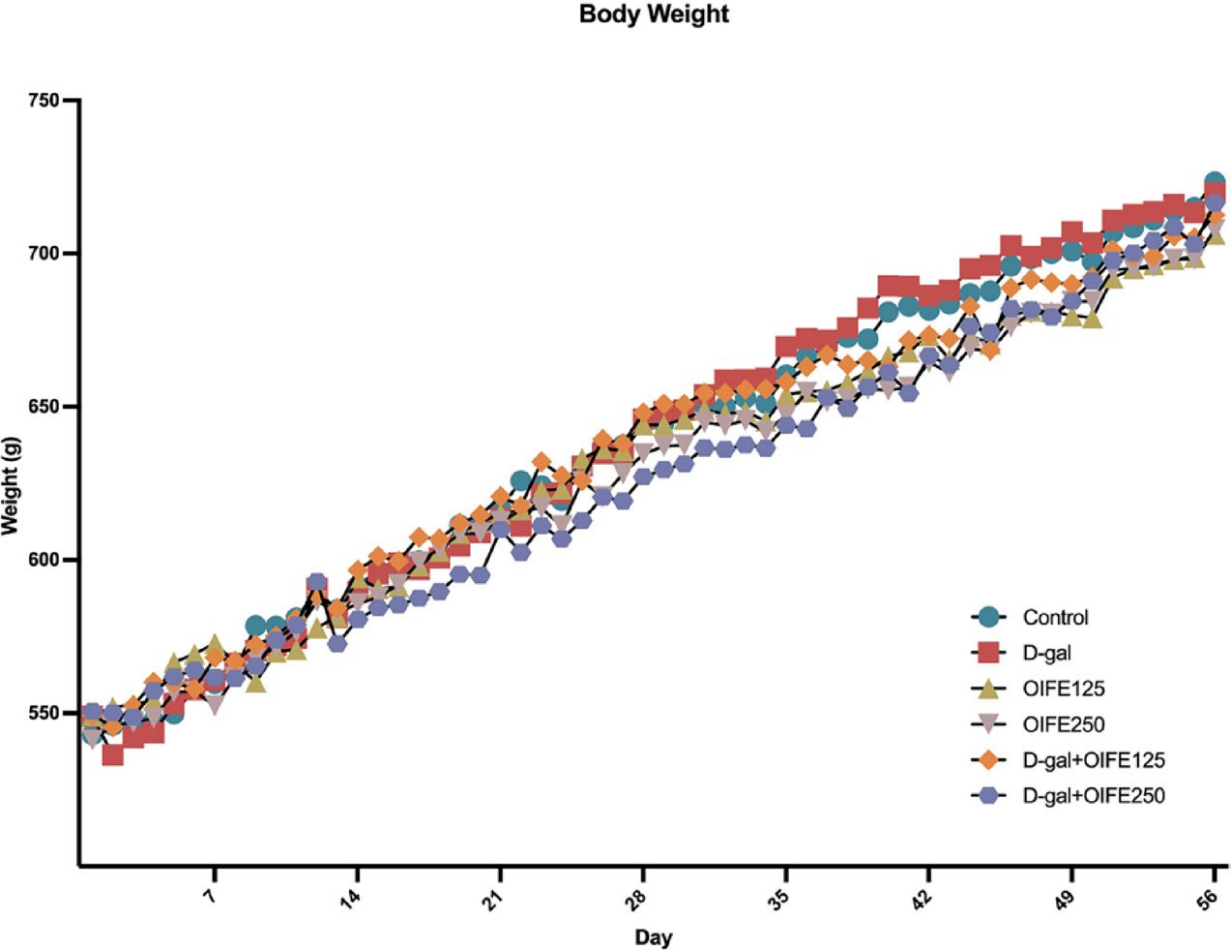
Impact of OIFE and D-gal on body weight**.**

**Fig. 3:**
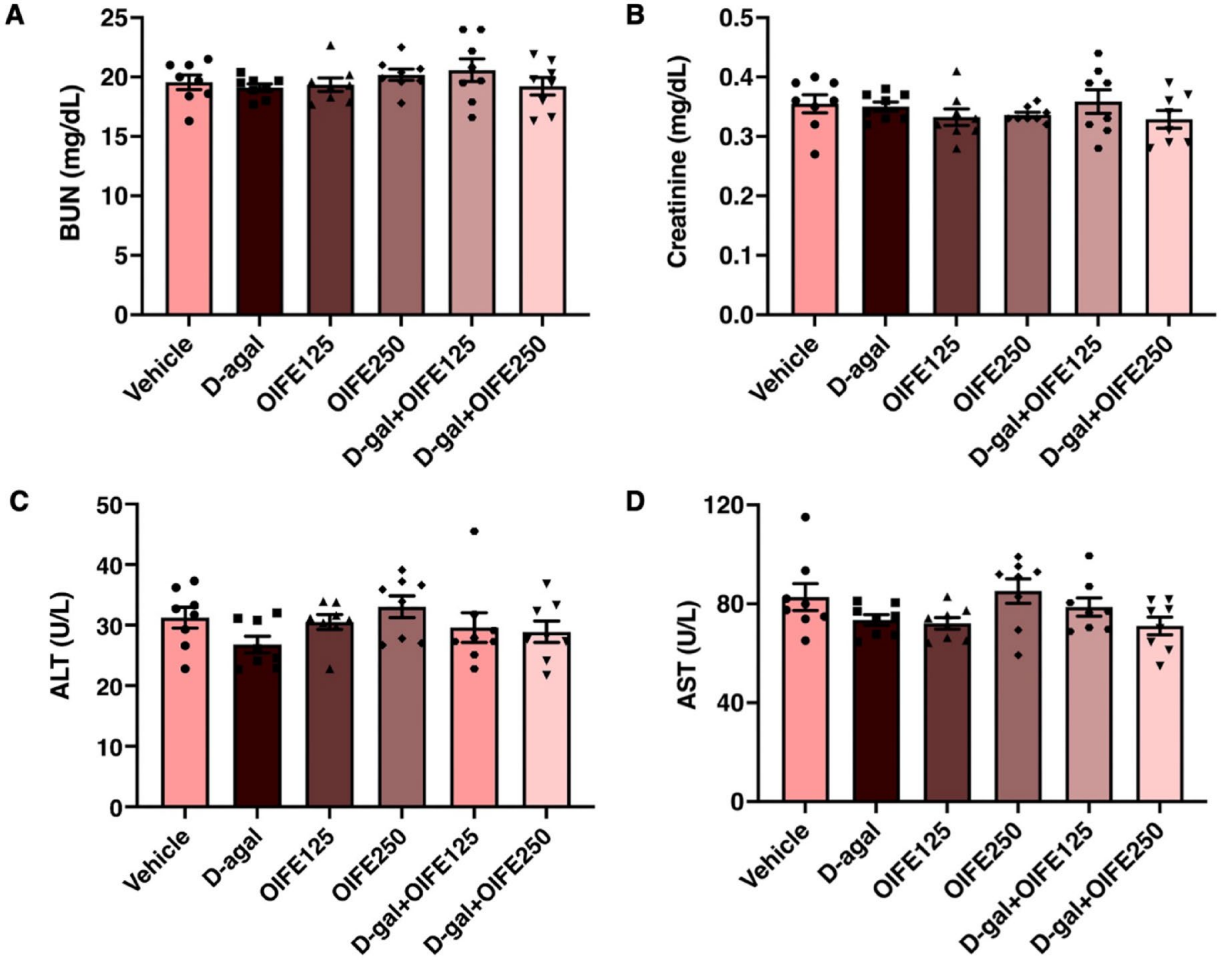
The effect of OIFE on kidney and liver function tests, as determined through biochemical investigations, including (**A**) BUN, (**B**) Creatinine, (**C**) ALT, and (D) AST.

No significant differences were observed in the animal body weight among the groups (F (275, 2640) = 0.9873, *p* = 0.547, effect size (η^2^) = 0.093), as shown in Fig. 2. The effect of OIFE on kidney and liver function tests, as determined through biochemical investigations, revealed no significant differences in blood urea nitrogen (BUN) (F (5, 42) = 0.841, *p* = 0.529, effect size (η^2^) = 0.091), creatinine (F (5, 42) = 0.853, *p* = 0.521, effect size (η^2^) = 0.092), alanine aminotransferase (ALT) (F (5, 42) = 1.456, *p* = 0.225, effect size (η^2^) = 0.148), and aspartate aminotransferase (AST) (F (5, 42) = 0.841, *p* = 0.06, effect size (η^2^) = 0.217), as presented in Fig. 3. The results indicate that the treatment with OIFE (125 and 250 mg/kg) and D-gal (50 mg/kg) did not result in any adverse events or major toxicity in kidney and liver functions across all groups.

### Effect of OIFE on memory functions

**Fig. 4:**
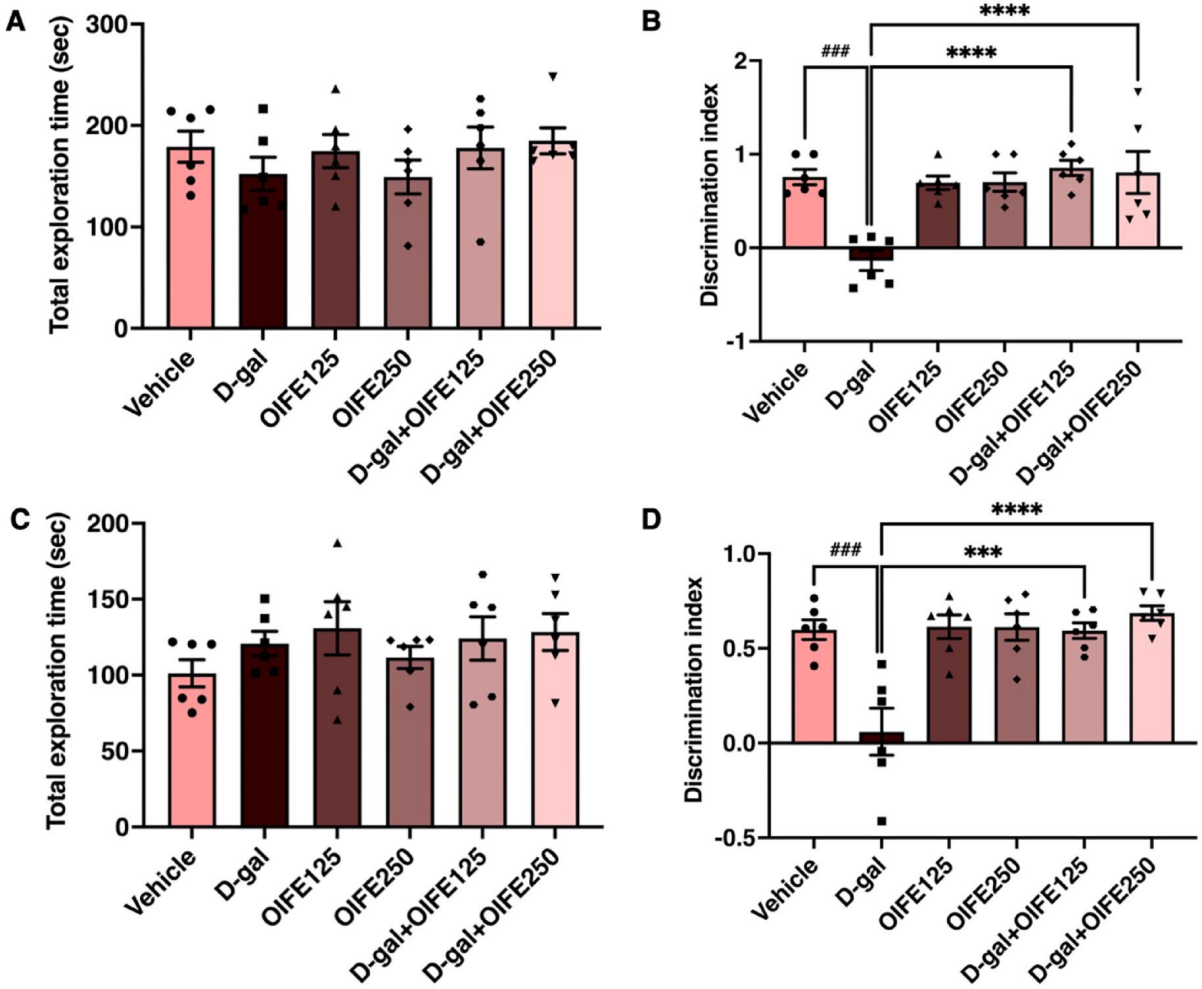
The impact of OIFE on D-gal-caused memory loss. The total exploration time of the NOL and NOR tests in the six groups (**A** and **C**). The discrimination index of the NOL and NOR tests in the six groups (**B** and **D**). Data are demonstrated as mean ± SEM. The markers ^#^ and ^*^ indicate comparisons with the vehicle and D-gal groups, respectively. Significance levels are set at ^###^ *p* < 0.001, ^***^ *p* < 0.001, and ^****^p < 0.0001.

The effects of OIFE and D-gal on memory function were assessed through the NOL and NOR tests, which measured spatial and recognition memory in D-gal-induced aging rats. There was no significant difference in exploration time across the groups in both the NOL (F (5, 30) = 0.827, *p* = 0.541, effect size (η^2^) = 0.121, Fig. 4A) and NOR tests (F (5, 30) = 0.888, *p* = 0.501, effect size (η^2^) = 0.129, Fig. 4C). These results suggest that neither OIFE nor D-gal treatment impacted the rat’s locomotion or movement. The discrimination indexes (DIs) of the D-gal group were significantly lower than those of the vehicle group (*p* < 0.0001, Fig. 4B and 4D), suggesting that D-gal impaired both spatial and recognition memory in the rats. Rats in the D-gal + OIFE125, and D-gal + OIFE250 groups showed significantly increased DIs in the NOL and NOR tests compared to the D-gal group [NOL test (F (5, 30) = 9.230,* p* < 0.0001, effect size (η^2^) = 0.606) and NOR test (F (5, 30) = 10.77, *p* < 0.0001, effect size (η^2^) = 0.642], signifying that OIFE can mitigate the impact of D-gal-induced brain aging as shown in Fig. 4 and Table 2.

**Table 2:** Mean the total exploration time and difference index of the novel object location (NOL) and novel object recognitions (NOR) test after treatment.

Group	Novel object location (NOL)		Novel object recognition (NOR)	
	Total exploration time (sec)	Discrimination index	Total exploration time (sec)	Discrimination index
Vehicle	179.0 ± 15.42	0.76 ± 0.08	101.1 ± 8.93	0.60 ± 0.05
D-gal	152.2 ± 16.45	-0.14 ± 0.11^###^	120.7 ± 8.06	0.06 ± 0.12^###^
OIFE125	174.6 ± 16.26	0.70 ± 0.07	130.8 ± 17.46	0.61 ± 0.06
OIFE250	149.2 ± 16.70	0.70 ± 0.10	111.6 ± 7.249	0.61 ± 0.07
D-gal + OIFE125	177.8 ± 20.56	0.85 ± 0.08****	124.1 ± 14.24	0.59 ± 0.04***
D-gal + OIFE250	184.7 ± 12.77	0.80 ± 0.22****	128.3 ± 12.10	0.70 ± 0.03****

^###^ *p* < 0.001 indicates comparison with the vehicle, and *** *p* < 0.001, and ****p < 0.0001 indicate comparisons with the D-gal groups (one-way ANOVA, Tukey’ s post-hoc test).

### Effect of D-gal and OIFE on immature neurons

**Fig. 5:**
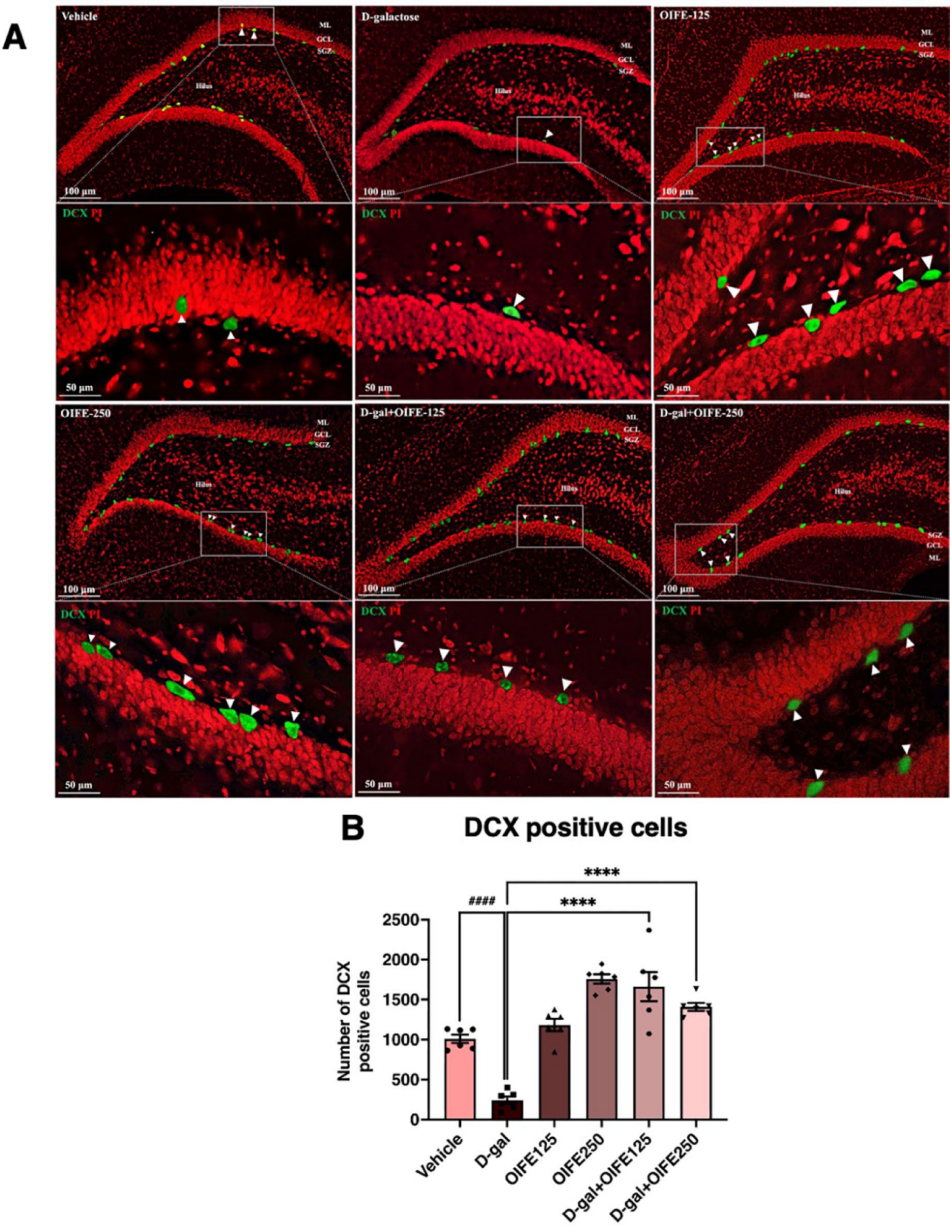
Immature neurons in the SGZ of the dentate gyrus in the hippocampus were assessed using DCX immunofluorescence staining. Green and red represent cells stained DCX and propidium iodide, respectively. White arrows represent DCX positive cells. Data are demonstrated as means ± SEM. The markers ^#^ and ^*^ indicate comparisons with the vehicle and D-gal groups, respectively. Significance levels are set at ^****^p < 0.0001 and ^####^*p* < 0.0001. SGZ: subgranular zone; GCL: granule cell layer; ML: molecular layer.

DCX immunofluorescence staining was applied to evaluate immature neurons in the SGZ as shown in Fig. 5. The mean number of DCX-positive cells were showed in Table S2. The D-gal group had significantly fewer cells stained with DCX compared to the vehicle group (*p* < 0.0001, Fig. 5B). However, a higher number of cells stained with DCX was detected in the co-treatment groups compared to the D-gal group (F5,30 = 36.51, *p* < 0.0001, effect size (η^2^) = 0.195), suggesting that OIFE can protect against the D-gal-induced reduction of immature neurons in the SGZ.

### Effect of D-gal and OIFE on and neuronal cell survival

**Fig. 6:**
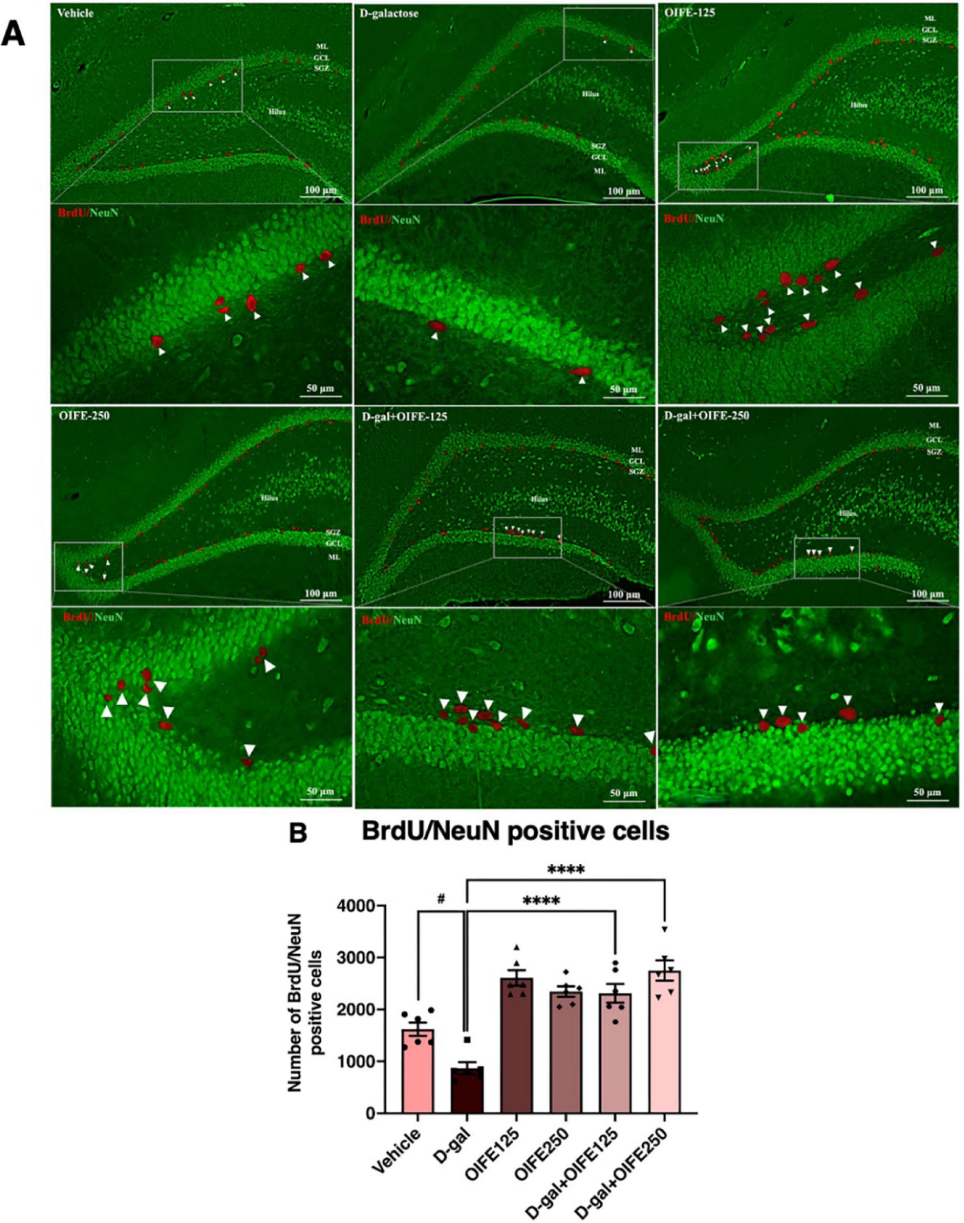
BrdU/NeuN double immunofluorescence investigation. Green and red represent cells stained BrdU and NeuN, respectively. White arrows represent DCX positive cells. Data are demonstrated as means ± SEM. The markers ^#^ and ^*^ indicate comparisons with the vehicle and D-gal groups, respectively. Significance levels are set at ^#^ *p* < 0.05 and ^****^ *p* < 0.0001. SGZ: subgranular zone; GCL: granule cell layer; ML: molecular layer.

Neuronal cell survival was assessed through double immunofluorescence staining of BrdU/NeuN as shown in Fig. 6. The mean number of BrdU/NeuN-positive cells were showed in Table S3. The D-gal group had significantly fewer cells stained BrdU/NeuN compared to the vehicle group (*p* < 0.05, Fig. 6B). However, treated with both the OIFE (125 mg/kg and 250 mg/kg) and D-gal ameliorated the reduction of neuronal cell survival, as indicated by cells stained BrdU/NeuN compared to the D-gal group (F5,30 = 22.93, *p* < 0.0001, effect size (η^2^) = 0.793, Fig. 6B). This suggests that OIFE can improve neuronal cell survival in D-gal-triggered brain aging.

## Discussion

The present study involved the quantification of phytochemicals in *O. indicum* fruit. The analysis showed an abundant quantity of flavonoids, phenols, as well as good antioxidant activity. HPLC analysis of *O. indicum* revealed several flavonoids particularly baicalein, oroxin A, chrysin, oroxylin A, and baicalin, which contributed to its observed free radical scavenging capacity. Various parts of *O. indicum* have been reported to exhibit potent antioxidant activity, attributable to their rich content of flavonoid compounds^22,24,25^. The antioxidant effects of OIFE are therefore primarily mediated by these flavonoids^23,29,30^. The present study showed the highest baicalein of OIFE, which acts as a potent antioxidant and anti-inflammation^31^. Numerous studies reported that its effects of baicalein have a good protective effect by decreasing oxidative stress and pro-inflammatory mediators on kidney, liver, and brain tissues^31–33^.

Consistent with a previous study that reported strong antioxidant activity and neuroprotective effects for *O. indicum* extract at (250–500 mg/kg)^26^, the 250 mg/kg dose was selected as the reference dose for this study. We included a low dose of 125 mg/kg to determine if OIFE’s efficacy is dose-dependent, given that the established effective rage (100–150 mg/kg), has previously yielded significant anti-inflammatory and antioxidant outcomes in rodent models^22^. In this animal study, co-administration of OIFE (125 mg/kg and 250 mg/kg) with D-gal (50 mg/kg) did not induce weight loss or kidney and liver toxicity in aging rats. In studies involving rats and mice, *O. indicum* has been administered at doses ranging from 5 to 5000 mg/kg without causing weight loss, toxicity signs, or mortality^34,35^. While there is no evidence to suggest that OIFE directly enhances neurogenesis, it can effectively protect against neuronal cell damage by increasing antioxidant activity. The effect of oxidative stress induces abnormality in various cells consequently causing cell injury. Oxidative stress arises from an imbalance between ROS and the antioxidant defense system, which is closely associated with decreasing neurogenesis and memory functions^36^. Furthermore, previous studies have demonstrated that the extraction of *O. indicum* can effectively prevent neuronal cell death induced by oxidative stress and inflammation, thereby contributing to improve memory and cognitive function^29,30^. Extracts of *O. indicum* can ameliorate the adverse effect of chemotherapy by providing antioxidant protection^26^. Accordingly, the ability of OIFE to restore hippocampal neurogenesis and ameliorate memory dysfunction may stem primarily from its antioxidant-mediated neuroprotective mechanisms.

The hippocampus is a key brain area involved in learning and memory, encompassing the process of information acquisition, encoding, consolidation, and retrieval. Dysfunction of the hippocampus is associated with memory impairment^37,38^. For the behavioral assessments, the NOL and NOR test were employed to investigate the neuroprotective effects of OIFE to assess spatial and recognition memory, respectively^39^. The NOL and NOR tests assess hippocampal and perirhinal cortex function by evaluating the rat’s spontaneous preference for exploring novel object locations and their recognition of previously encountered objects. These tests do not require reinforcement^40^. Previous studies have consistently shown that D-gal induces memory impairment in aging rat models, and our findings are in agreement with these observations^17,19,20^. Our results showed that the co-administration of D-gal and OIFE extract significantly improved spatial and recognition memory. Notably, previous research in animal models of aging has reported that co-treatment with chrysin or baicalin (both components found in OIFE) can mitigate memory deficits and restore hippocampal neurogenesis impaired by D-gal or valproic acid^19,41^. Therefore, the observed protective effect of OIFE against D-gal-induced memory impairment, as indicated by the NOL and NOR test results, is likely mediated by these bioactive flavonoids.

A variety of stimuli can disrupt hippocampal neurogenesis, promoting to the development of brain disorders. Impairment of hippocampal neurogenesis leads to learning and memory dysfunction, potentially by decreasing cell survival. Under healthy conditions, hippocampal neurogenesis involves in the differentiation of neural stem cells into immature neurons, characterized by the expression of DCX, a microtubule-associated protein expressed during the early stage of differentiation^42^. A previous study reported that *O. indicum* extract particularly its major flavonoids enhances neuronal differentiation, possibly by activating the neurogenin2 (Ngn2) promoter and altering of C17.2 neural stem cell fate^43^. Our study showed that co-administration of OIFE can restore the population of immature neurons in the SGZ. These immature neurons subsequently mature and integrate into existing neural circuits, as evidenced by BrdU incorporation and co-localization with neuronal nuclei antigen (NeuN), a marker of mature post-mitotic neurons. BrdU, a thymidine analog incorporated into newly synthesized DNA, serves as a marker of cell proliferation and survival^44^. Co-localization of BrdU and NeuN is used to identify mature neurons that have originated through neurogenesis and survived^19,20,45^. In the present study, BrdU/NeuN co-localization revealed that chronic D-gal administration significantly decreased neuronal cell survival, indicative of brain aging. However, OIFE treatment recovered hippocampal neurogenesis by increasing the number of surviving cells in the SGZ. D-gal accumulation is known to increase ROS generation through the activity of galactose oxidase, leading to the production of hydrogen peroxide (H_2_O_2_) and hydroxide (OH^−^). This disruption of redox homeostasis contributes to increased inflammation and impaired neurogenesis, ultimately resulting in neuronal cell damage^16,46^. The flavonoid constituents of OIFE are proposed to act as an electron donor, exhibiting free radical scavenging activity, thereby contributing to redox homeostasis and potentially reducing inflammation^47^. Additionally, the results of the present study align with previous studies reported that *O. indicum* extract exhibits antioxidant and anti-inflammatory activities in BV2 microglial cells^30^ and protects SH-SY5Y cells against β-amyloid-induced cell injury by mitigating oxidative stress, suppressing neuroinflammation, and promoting cell survival pathways^29^. Overall, OIFE co-treatment may mitigate D-gal-induced oxidative stress through its antioxidant and neurotrophic mechanisms, thereby ameliorating D-gal-induced neurogenesis impairment and memory deficits in aging rat brains.

Our findings align with previous research demonstrating that oroxylin A, a compound in OIFE, increases neuronal cell proliferation and survival, thereby enhancing neurogenesis via increased synaptic plasticity and upregulation of brain-derived neurotrophic factor (BDNF) and the transcription factor cyclic adenosine monophosphate response element binding protein (CREB)^48,49^. In agreement with these mechanisms, a recent study on *O. indicum* extract reported up-regulation of BDNF gene expression in neuronal cells exposed to inflammatory stimuli and improved cognitive outcomes in people with mild cognitive impairment^27^. Baicalein, which is also present in OIFE, decreased neuronal cell inflammation, increased cell survival, and attenuated sevoflurane-induced behavioral, learning, and memory impairments in rats^50^. Consistent evidence suggests that baicalein protects the hippocampal neurogenic niche and enhances cognition by exerting both antioxidant and neurotrophic actions^51^. In an irradiation model, baicalein attenuated ROS accumulation, preserved neural stem cell viability, restored neurogenesis in the dentate gyrus, and prevented spatial memory deficits and enhanced BDNF/pCREB signaling^52^. Baicalein and baicalin have also been reported to activate the nuclear factor erythroid 2–related factor 2 (Nrf2)/antioxidant response element (ARE) signaling pathway, leading to an increase in antioxidant enzymes such as induce the expression of heme oxygenase-1 (HO-1), superoxide dismutase (SOD), catalase (CAT), and glutathione peroxidase (GPx). These enzymes subsequently reduce oxidative stress and promote neuronal survival^31,53–55^. These pathways maintain redox homeostasis and protect hippocampal progenitor cells from ROS-induced senescence, thereby promoting neuronal differentiation and synaptic plasticity. Both compounds also enhanced BDNF expression and extracellular signal-regulated kinases (ERK), and CREB signaling, which play crucial roles in neurogenesis and cognitive function^23,31,50,56^. Baicalin, a major flavonoid in OIFE, counteracted valproic acid-induced memory deficits and impaired neurogenesis in adult rats by restoring Ki-67/RECA-1, BrdU, and DCX positive cell counts and improving spatial and recognition memory^41^. Chrysin and oroxin A supplementation protects against D-gal-induced impairments in hippocampal neurogenesis and memory in brain aging^19,48,49^. Moreover, chrysin has also been demonstrated to suppress oxidative stress, p21-mediated cell-cycle arrest, and neuronal apoptosis. This action restores hippocampal neurogenesis and improves memory in D-galactose-induced aging rat models^57,58^.

In conclusion, OIFE demonstrated potent antioxidant and neuroprotective effects, successfully ameliorating D-gal–induced memory deficits and neurogenesis impairments in rats. These actions are likely ameliorated by its abundant flavonoid and phenolic constituents, which counteract oxidative stress and prevent neuronal damage associated with brain aging. Co-treatment with OIFE resulted in an increase in the number of both immature and mature neurons, correlating with improved spatial and recognition memory. Although our findings provide preclinical evidence supporting OIFE’s beneficial effects, further mechanistic and translational studies are needed to elucidate its underlying pathways and confirm its therapeutic relevance. Therefore, OIFE may be considered a potential natural compound for further investigation to prevent or mitigate age-related cognitive decline.

## Methods

### Chemicals and reagent

The fresh fruit of *O. indicum* was collected between June and December from a local market in Muang Khon Kaen District, Khon Kaen, Thailand in 2022. The following chemicals: gallic acid, Folin–Ciocalteau reagent, quercetin, aluminum chloride, 2,2-diphenyl-1-picryl-hydrazyl (DPPH), L-ascorbic acid, and D-galactose were acquired from Sigma Chemical Company (St. Louis, MO, USA). Moreover, anti-BrdU antibody, anti-NeuN antibody, and normal goat serum were acquired from Abcam Limited (Abcam, Cambridge, UK). Alexa Fluor 568 goat anti-rabbit IgG and Alexa Fluor 488 rabbit anti-mouse IgG were acquired from Thermo Fisher Scientific (Invitrogen, Carlsbad, CA, USA).

### Ethical approval

All animal experiments of this study were approved by the Khon Kean University Ethics Committee in Animal Research (Record No. IACUC-KKU-71/65, Date of Approval: 18 August 2022) and were performed in accordance with the Ethic of Animal Experimentation of National Research Council of Thailand and ARRIVE guidelines thereof.

### Preparation of O. indicum fruits extract (OIFE)

The *O. indicum* fruit was prepared following a previously published method^29^. The plant samples were collected from the local market at Meaung Khon kaen district, Khon Kaen Province, Thailand. The voucher specimen (*N. Tanrangka 1* [KKU No. 26744]) was deposited at the flora of Khon Kaen University (KKU) Herbarium and authenticated by Dr. Natthawut Triyutthachai, a plant taxonomist at the Department of Biology, Facullty of Science, KKU, Thailand. Selected mature and undamaged *O. indicum* fruits were washed with tap water to remove contaminants. Chopped *O. indicum* fruit was dried and weighed, followed by static maceration in 95% (v/v) ethanol for 7 days at room temperature. Then, OIFE was filtered, concentrated, and freeze-dried. The production of OIFE was 13.51% per dry weight of *O. indicum* fruits.

### Phytochemical analyses

#### Determination of total phenolic content (TPC) assay

A stock solution of OIFE was prepared and gallic acid was used for calibration. The mixtures of Folin–Ciocalteau reagent and sodium carbonate solution were combined with the OIFE and gallic acid solutions, followed by incubating in darkness for 2 h at room temperature and assessing in triplicate. The optical density was measured at 750 nm using a spectrophotometer (Evolution™300 UV–Vis Spectrophotometer, Thermo Fisher Scientific, Darmstadt, Germany) and the total phenolic element in the OIFE was manifested as mg of gallic acid equivalents (GAE) per gram of OIFE^59^.

#### Measurement of total flavonoid content (TFC) assay

The aluminum chloride colorimetric method was employed to determine the total flavonoid content in the OIFE, using quercetin as the reference material, as adapted from earlier research^59^. After incubating at room temperature, the mixture of the OIFE, a spectrophotometer was used to quantify the TFC at 430 nm and the TFC assay was performed in triplicate. TFC content in the OIFE was manifested as mg quercetin equivalent (QE) per gram of the OIFE.

#### Measurement of free-radical-scavenging activity 2,2-diphenyl-1-picryl-hydrazyl (DPPH) radical scavenging activity

The DPPH radical scavenging activity method was modified to quantify the free radical scavenging ability in the OIFE and with L-ascorbic acid as the standard^60^. Equal volumes of OIFE or L-ascorbic acid solution and DPPH solution were mixed and incubated in the darkness for 30 min at room temperature. After incubating, the optical density was quantified at 517 nm and the DPPH radical scavenging activity was performed in triplicate. The radical scavenging capacity was evaluated to estimate the IC_50_ values necessary to scavenge 50% of DPPH free radicals.

#### Measurement of ferric reducing antioxidant power (FRAP)

The ferric reducing antioxidant power (FRAP) method was utilized to determine the total antioxidant power in the OIFE by evaluating its ability to reduce a ferric tripyridyltriazine (Fe^3+^-TPTZ) complex to its ferrous (Fe^2+^) form at low pH^61^. The FRAP reagent was combined and incubated with the OIFE or L-ascorbic acid for 10 min at 37 °C and the FRAP measurement was performed in triplicate. After that, the optical density was quantified at 593 nm and the FRAP value of the OIFE was determined using a calibration curve of L-ascorbic acid and manifested as µM L-ascorbic acid equivalent per mg of the OIFE.

#### High performance liquid chromatography (HPLC)

The OIFE was standardized using HPLC connected to ultraviolet wavelength detector (HPLC–UV) for analysis. Quantitative HPLC assessment was conducted using a SHIMADZU i-Series system with separated achieved a Purospher® STAR RP-18 column HPLC columns (5 µm) for polarity separation of chemical molecules as described and conducted previously by Choonong et al.^28,62^. The gradient mobile phase of HPLC system used acetonitrile and 0.05% phosphoric acid in water with 0.8 ml/min flow rate. Briefly, the samples of OIFE were sampling and prepared in absolute ethanol until dissolved, filtered through 0.45 μm filter to protect column obstruction, and compared to standard solutions of cinnamic acid, baicalein, baicalin, oroxylin A, oroxin A, and chrysin (Sigma Aldrich, St. Louis, USA). The samples 10 and 1 mg/ml of OIFE were injected at 20 μL per injection in triplicate (n = 3). Detection was carried out at 280 nm and peak areas were calculated using the triangular method to create calibration curves for quantification. The method validation of HPLC was described in previous study^28^ as the standard curve of oroxin A, cinnamic acid and chrysin were used in range of 0.78–100 µg/ml, baicalin and oroxylin A were used in range of 1.56–100 µg/ml and baicalein was used in range of 6.25–100 µg/ml. All of standard were prepared from 100 µg/ml and used two-folds dilution until lowest concentration for using 5–7 concentrations depending on each standard compound. The linearity, limit of detection (LOD), and limit of quantification (LOQ) were described in previous study^28^. The precision was described to intra-day and inter-day precision (n = 3) for 5 concentrations were 0.75, 6.25., 12.5, 25.0 and 100 μg/ml which was calculated to relative standard deviation (RSD)^28^.

### Animal and experimental design

**Fig. 7:**
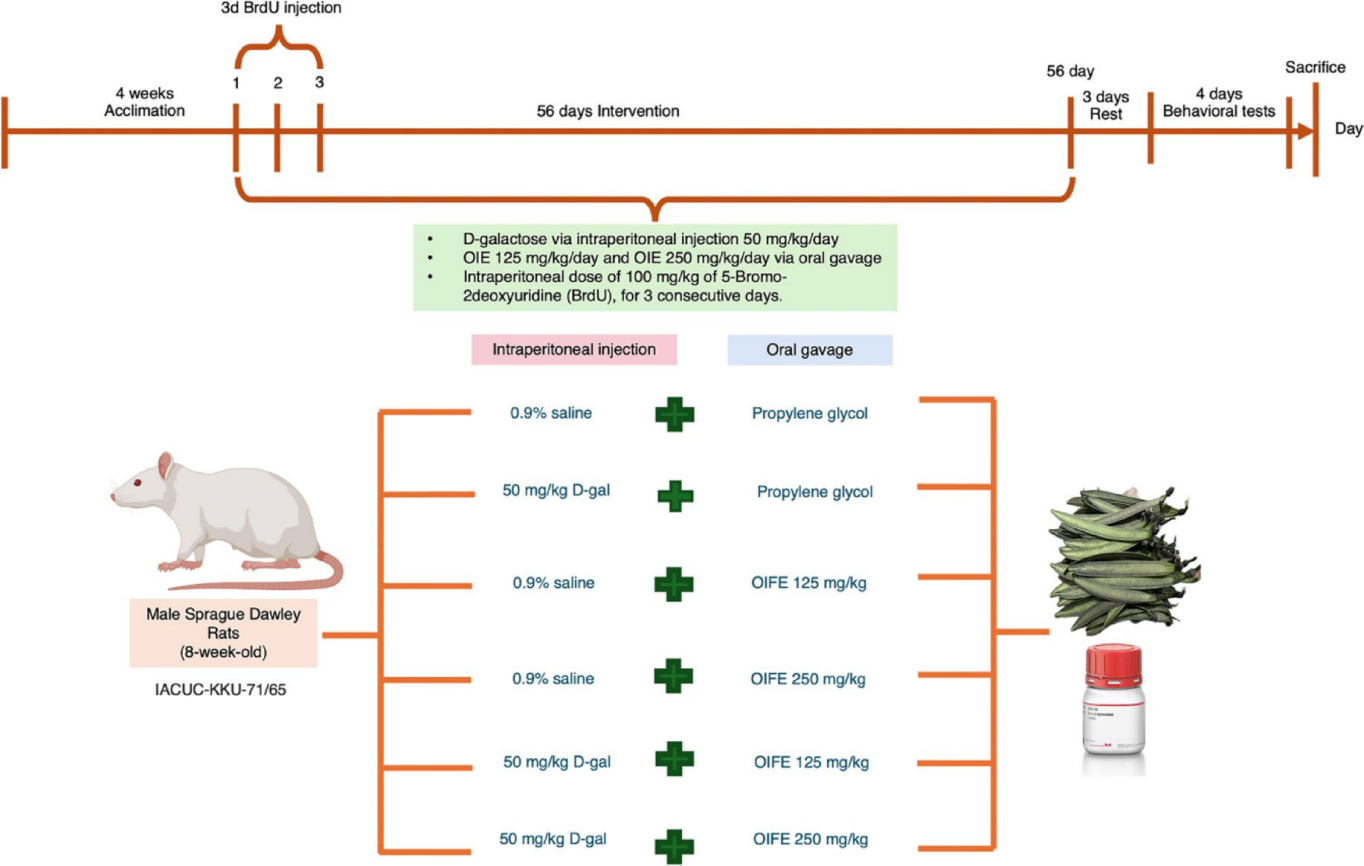
The diagram illustrates the timeline of treatment and the experimental animal groups.

This experiment involved 60 adult male Sprague–Dawley rats, aged 8 weeks and weighing between 280–300 g, obtained from Nomura Siam International Co., td. Pathumtawan, Bangkok. Three to four rats were allocated per cage. The rats were provided with food and water, maintained at a temperature of 25 °C and kept on a 12-h light/dark cycle. The research guidelines were authorized by the Institutional Animal Care and Use Committee of Khon Kaen University (IACUC-KKU-71/65) and ARRIVE guidelines thereof. After 4-week of acclimation, the rats were divided into six groups (10 rats per group): vehicle, D-gal, OIFE-125, OIFE-250, D-gal + OIFE125, and D-gal + OIFE250. The rats received D-gal at a dose of 50 mg/kg via daily intraperitoneal injection for 56 days. Additionally, OIFE was administered via daily oral gavage for 56 days at doses of 125 mg/kg and 250 mg/kg with propylene glycol. The rats underwent a 3-day rest period prior to behavioral testing to minimize residual stress from drug administration, handling, or related procedures. Memory alterations were determined utilizing novel object location (NOL) and novel object recognition (NOR) examinations after drug administration. Before starting the experiment, all groups of rats received an intraperitoneal dose of 100 mg/kg of BrdU, a marker for studying cell survival, dissolved in 0.9% saline solution for 3 consecutive days. The timeline of animal experiment was shown in Fig. 7.

### Behavioral tests

The NOL and NOR tasks were modified from previous published protocols^19,63^. In the NOL and NOR tests, rats were habituated to an empty arena 24 h before testing to minimize context-related novelty and anxiety, ensuring that subsequent exploration reflects object novelty and/or location rather than the arena context. Data from both tests were recorded using EthoVision® XT software (version 12, Noldus, Wageningen, Netherlands) The framework apparatus for all behavioral tasks was composed of an arena (50 × 50 × 50 cm, width x length x high) and round plastic bottles filled with red water (1000 ml capacity; 72 mm diameter × 265 mm height). Video tracking data for both tests were recorded with EthoVision® XT software (Etho Vision ®, XT version 12, Noldus, Wageningen, Netherlands). For the NOL test, the rats were habituated to a vacant arena for 30 min one day before the test day. The following day, rats were again habituated for 3 min. The next day, they were habituated again for 3 min before being introduced to two similar objects in the arena for 3 min during the familiarization trial. After a 15-min break, the rats then were given the opportunity to explore the objects placed in novel and familiar locations for 3 min, and their exploration time was recorded to calculate the discrimination index^19,40,63^. In the NOR test, the rats were allowed to freely explore the arena for 30 min during the habituation. The next day, they were re-habituated for 3 min and exposed two similar objects in the arena for 3 min during familiarization phase. After a 15-min break, the rats were permitted to investigate both the familiar object and a novel object for 3 min. The arena and objects were cleaned with 20% ethanal to reduce the olfactory cues. The DI was then worked out based on their exploration time as outlined in previous studies^19,40,63^. The DI was calculated as: (Novel object exploration time − Familiar object exploration time) / (Novel object exploration time + Familiar object exploration time)^64^.

### Kidney and liver function measurement

After the behavioral tests, blood samples were collected from the lateral tail vein to assess the impact of OIFE on liver and kidney function tests. These analyses were performed by Clinical Chemistry and Immunology Unit, Clinical Laboratory Section, Srinagarind Hospital, Faculty of Medicine, Khon Kaen University to obtain serum biochemical parameters, including blood urea nitrogen (BUN), creatinine, alanine aminotransferase (ALT), and aspartate aminotransferase (AST). These tests were measured using an automated clinical chemistry analyzer (cobas c701 module, Roche Diagnostics GmbH, Mannheim, Germany) with commercial reagent kits (ASTP2, ALTP2, CREP2, UREAL; Roche Diagnostics). BUN was measured by the enzymatic urease– glutamate dehydrogenase (GLDH) method, based on NADH consumption at 340 nm. Creatinine was analyzed by the enzymatic method involving creatininase, creatinase, sarcosine oxidase, and peroxidase (Trinder reaction), with quinoneimine dye formation measured at 550 nm^65^. AST and ALT were analyzed using the kinetic methods recommended by the International Federation of Clinical Chemistry and Laboratory Medicine (IFCC), based on NADH consumption at 340 nm^66^. The results were expressed as mg/dL for BUN and creatinine, and U/L for AST and ALT.

### Immunofluorescence

After the behavioral assessments, the rats were euthanized by cervical dislocation and decapitation. Anesthetic agents were not used in this study. The brain tissues were collected for immunofluorescence analysis modified from previous published protocols^19,63^. One hemisphere of the brain was fixed in 30% sucrose cryoprotective solution for 4 h at 4 °C to prevent tissue damage from freezing. It was subsequently embedded in optimal cutting temperature compound (Themo Fisher Scientific, Karlsruhe, Germany). The brain hemispheres were then frozen in isopentane cooled with liquid nitrogen (Sigma-Aldrich, Inc., St. Louis, USA) and stored at − 80 °C for immunofluorescence staining. Thereafter, the brain hemispheres were sectioned with a thickness of 40 µm along the coronal plane using a cryostat (Cryostat Series HM 550 Microm International; Walldorf, Germany). Every eighth section throughout the full length of the dentate gyrus (9 sections per brain) were selected for BrdU/NeuN and DCX immunofluorescence staining. For BrdU/NeuN assessment, the sections were incubated with anti-BrdU (1:100, Abcam, Cambridge, UK) primary antibody at 4 °C overnight. On the second day, the sections were incubated with a secondary antibody [Alexa Fluor 568 goat anti-rabbit IgG (1:400, Invitrogen, Carlsbad, CA, USA)] for 60 min, followed by incubation with primary anti-NeuN antibody (1:500, Sigma Aldrich, St. Louis, USA) at 4 °C overnight. On the third day, the sections were incubated with a secondary antibody [Alexa Fluor 488 rabbit anti-mouse IgG (1:500, Invitrogen, Carlsbad, CA, USA)] for 60 min and then mounted using glycerol. For DCX straining, the sections were incubated with a primary anti-DCX antibody (1:100, Santa Cruz. USA) at 4 °C overnight. On the second day, the sections were incubated with a secondary antibody (Alexa Fluor 488 rabbit anti-mouse IgG (1:500, Invitrogen, Carlsbad, CA, USA)) for 60 min, followed by a counterstain with propidium iodide and mounting using glycerol.

BrdU/NeuN and DCX-stained cells were counted within a distance equivalent to three cell bodies in the SGZ using a Nikon Eclipse 80i fluorescence microscope. The total number of stained cells was calculated by summing the counts from 9 sections per brain, and then the numbers of BrdU/NeuN and DCX-positive cell counts were multiplied by 8^19,39,63^.

### Statistical

All statistical evaluations were performed using Graphpad Prism (Version 10; Graphpad Software Inc., San Diego, CA, USA). The data are represented as mean ± standard error of mean (SEM). Statistical examinations were conducted using two-way analysis of variance (ANOVA) for animal body weight (in gram), and one-way ANOVA followed by Tukey’s multiple comparison test for the total exploring time, the DI values, biochemical analysis, phytochemical assessments, the counts of BrdU/NeuN and DCX. All statistical evaluations at the probability level of *p* < 0.05 were statistically significant.

## Supplementary Information

Below is the link to the electronic supplementary material.

Supplementary Material 1

## Data Availability

All data generated or analyzed during this study are included in this published article.

## References

[1] UN, D. World population prospects: the 2012 revision. *UN Department of Economic and Social Affairs* (2013).

[2] Peters, R. Ageing and the brain. *Postgrad. Med. J.* **82**, 84–88 (2006). 16461469 10.1136/pgmj.2005.036665 PMC2596698

[3] Lee, J. & Kim, H.-J. Normal aging induces changes in the brain and neurodegeneration progress: Review of the structural, biochemical, metabolic, cellular, and molecular changes. *Fron. Aging Neurosci.* **14**, 931536 (2022). 10.3389/fnagi.2022.931536 PMC9281621 35847660

[4] Colita, E., Mateescu, V. O., Olaru, D.-G. & Popa-Wagner, A. Cognitive decline in ageing and disease: Risk factors, genetics and treatments. *Curr. Health Sci. J.* **50**, 170–180 (2024). 39371061 10.12865/CHSJ.50.02.02 PMC11447500

[5] Culig, L., Chu, X. & Bohr, V. A. Neurogenesis in aging and age-related neurodegenerative diseases. *Ageing Res. Rev.* **78**, 101636 (2022). 35490966 10.1016/j.arr.2022.101636 PMC9168971

[6] Babcock, K. R., Page, J. S., Fallon, J. R. & Webb, A. E. Adult hippocampal neurogenesis in aging and Alzheimer’s disease. *Stem Cell Rep.* **16**, 681–693 (2021). 10.1016/j.stemcr.2021.01.019 PMC8072031 33636114

[7] Kumar, A., Pareek, V., Faiq, M. A., Ghosh, S. K. & Kumari, C. Adult neurogenesis in humans: A review of basic concepts, history, current research, and clinical implications. *Innov. Clin. Neurosci.* **16**, 30–37 (2019). 31440399 PMC6659986

[8] Toda, T., Parylak, S. L., Linker, S. B. & Gage, F. H. The role of adult hippocampal neurogenesis in brain health and disease. *Mol. Psychiatry* **24**, 67–87 (2019). 29679070 10.1038/s41380-018-0036-2 PMC6195869

[9] Baptista, P. & Andrade, J. P. Adult hippocampal neurogenesis: regulation and possible functional and clinical correlates. *Front. Neuroanat.* **12**, 44 (2018). 29922131 10.3389/fnana.2018.00044 PMC5996050

[10] Cui, X. et al. Chronic systemic D-galactose exposure induces memory loss, neurodegeneration, and oxidative damage in mice: protective effects of R-alpha-lipoic acid. *J. Neurosci. Res.* **83**, 1584–1590 (2006). 16555301 10.1002/jnr.20845

[11] Cohen, A. A. Aging across the tree of life: The importance of a comparative perspective for the use of animal models in aging. *Biochim. Biophys. Acta (BBA) Mol. Basis Dis.* **1864**, 2680–2689 (2018). 10.1016/j.bbadis.2017.05.028 28690188

[12] Azman, K. F. & Zakaria, R. D-Galactose-induced accelerated aging model: an overview. *Biogerontology* **20**, 763–782 (2019). 31538262 10.1007/s10522-019-09837-y

[13] Brito, D. V. C. et al. Assessing cognitive decline in the aging brain: lessons from rodent and human studies. *npj Aging* **9**, 23 (2023). 37857723 10.1038/s41514-023-00120-6 PMC10587123

[14] Banji, D., Banji, O. J. F., Dasaroju, S. & Kranthi, K. C. H. Curcumin and piperine abrogate lipid and protein oxidation induced by D-galactose in rat brain. *Brain Res.* **1515**, 1–11 (2013). 23566814 10.1016/j.brainres.2013.03.023

[15] Ahmad, S. et al. Fisetin rescues the mice brains against D-galactose-induced oxidative stress Neuroinflammation and Memory Impairment. *Front. Pharmacol.* **12**, 612078 (2021). 33716741 10.3389/fphar.2021.612078 PMC7947859

[16] Shwe, T., Pratchayasakul, W., Chattipakorn, N. & Chattipakorn, S. C. Role of D-galactose-induced brain aging and its potential used for therapeutic interventions. *Exp. Gerontol.* **101**, 13–36 (2018). 29129736 10.1016/j.exger.2017.10.029

[17] Nam, S. M. et al. Ascorbic acid mitigates D-galactose-Induced brain aging by increasing hippocampal neurogenesis and improving memory function. *Nutrients* **11**, 176 (2019). 30650605 10.3390/nu11010176 PMC6356429

[18] Sadigh-Eteghad, S. et al. D-galactose-induced brain ageing model: A systematic review and meta-analysis on cognitive outcomes and oxidative stress indices. *PLoS ONE* **12**, e0184122 (2017). 28854284 10.1371/journal.pone.0184122 PMC5576729

[19] Prajit, R. et al. Chrysin protects against memory and hippocampal neurogenesis depletion in D-galactose-induced aging in rats. *Nutrients* **12**, 1100 (2020). 32316121 10.3390/nu12041100 PMC7230764

[20] Saenno, R. et al. Caffeic acid alleviates memory and hippocampal neurogenesis deficits in aging rats induced by D-galactose. *Nutrients* **14**, 2169 (2022). 35631310 10.3390/nu14102169 PMC9145046

[21] Hengpratom, T. et al. Oroxylum indicum (L.) Kurz extract inhibits adipogenesis and lipase activity in vitro. *BMC Complement Altern. Med.* **18**, 177 (2018). 29884167 10.1186/s12906-018-2244-3 PMC5994072

[22] Jagetia, G. C. A review on the medicinal and pharmacological properties of traditional ethnomedicinal plant sonapatha *Oroxylum indicum*. *Sinusitis* **5**, 71–89 (2021).

[23] Nik Salleh, N. N. H., Othman, F. A., Kamarudin, N. A. & Tan, S. C. The Biological Activities and Therapeutic Potentials of Baicalein Extracted from *Oroxylum indicum*: A Systematic Review. *Molecules* **25**, 5677 (2020). 33276419 10.3390/molecules25235677 PMC7730069

[24] Dinda, B., SilSarma, I., Dinda, M. & Rudrapaul, P. Oroxylum indicum (L.) Kurz, an important Asian traditional medicine: From traditional uses to scientific data for its commercial exploitation. *J Ethnopharmacol.* **161**, 255–278 (2015). 25543018 10.1016/j.jep.2014.12.027

[25] Kang, I. N., Nik Salleh, N. N. H., Chung, W. J., Lee, C. Y. & Tan, S. C. Baicalein-enriched fraction extracted from *Oroxylum indicum* (L.) Benth. ex Kurz Leaves exerts antioxidant and inhibitory effects against glioblastoma multiforme. *Processes* **7**, 963 (2019).

[26] Pondugula, S. R. et al. *Oroxylum Indicum* ameliorates chemotherapy induced cognitive impairment. *PLoS ONE* **16**, e0252522 (2021). 34081735 10.1371/journal.pone.0252522 PMC8174701

[27] Lopresti, A. L., Smith, S. J., Majeed, M. & Drummond, P. D. Effects of an *Oroxylum indicum* extract (Sabroxy®) on cognitive function in adults with self-reported mild cognitive impairment: A randomized, double-blind, Placebo-Controlled Study. *Front. Aging Neurosci.* **13**, 728360 (2021). 34531736 10.3389/fnagi.2021.728360 PMC8438240

[28] Choonong, R. et al. Anti-inflammatory potential of *Oroxylum indicum* flavonoids: Effects of traditional grilling on aglycone flavonoid content and activity against urban dust-induced inflammation. *Food Biosci.* **62**, 105523 (2024).

[29] Mairuae, N., Connor, J., Buranrat, B. & Lee, S. *Oroxylum indicum* (L) extract protects human neuroblastoma SH-SY5Y cells against β-amyloid-induced cell injury. *Mol. Med. Rep.* **20**, 1933–1942 (2019). 31257498 10.3892/mmr.2019.10411

[30] Mairuae, N., Cheepsunthorn, P. & Buranrat, B. Antioxidant and anti-inflammatory activities of Oroxylum indicum Kurz (L.) fruit extract in lipopolysaccharide-stimulated BV2 microglial cells. *Trop. J. Pharm Res* **20**, 833–838 (2022).

[31] Si, L., An, Y., Zhou, J. & Lai, Y. Neuroprotective effects of baicalin and baicalein on the central nervous system and the underlying mechanisms. *Heliyon* **11**, e41002 (2025). 39758400 10.1016/j.heliyon.2024.e41002 PMC11699331

[32] Sahu, B. D., Kumar, J. M. & Sistla, R. Baicalein, a bioflavonoid, prevents cisplatin-induced acute kidney injury by up-regulating antioxidant defenses and down-regulating the MAPKs and NF-κB pathways. *PLoS ONE* **10**, e0134139 (2015). 26222683 10.1371/journal.pone.0134139 PMC4519041

[33] Zhou, H.-C. et al. Hepatoprotective effect of baicalein against acetaminophen-induced acute liver injury in mice. *Molecules* **24**, 131 (2018). 30602693 10.3390/molecules24010131 PMC6337302

[34] Farhan Hanif Reduan, M. et al. Acute oral toxicity study of ethanol extract of *Oroxylum indicum* leaf in mice. *Thai J. Vet. Med.* **50**, 573–581 (2020).

[35] Othman, F. A., Mat Zin, A. A., Zakaria, Y., Nik Salleh, N. N. H. & Tan, S. C. Dataset of acute oral toxicity and subacute neurotoxicity risk assessments of flavonoid-enriched fraction extracted from *Oroxylum Indicum* on sprague dawley rats. *Data Brief* **49**, 109411 (2023). 37520653 10.1016/j.dib.2023.109411 PMC10374857

[36] Franzoni, F. et al. Oxidative stress and cognitive decline: The neuroprotective role of natural antioxidants. *Front. Neurosci.* **15**, 729757 (2021). 34720860 10.3389/fnins.2021.729757 PMC8548611

[37] Wiltgen, B. J. et al. The hippocampus plays a selective role in the retrieval of detailed contextual memories. *Curr. Biol.* **20**, 1336–1344 (2010). 20637623 10.1016/j.cub.2010.06.068 PMC2928141

[38] Aimone, J. B. et al. Regulation and function of adult neurogenesis: From genes to cognition. *Physiol. Rev.* **94**, 991–1026 (2014). 25287858 10.1152/physrev.00004.2014 PMC4280160

[39] Umka Welbat, J. et al. Asiatic acid prevents the deleterious effects of valproic acid on cognition and hippocampal cell proliferation and survival. *Nutrients* **8**, 303 (2016). 27213437 10.3390/nu8050303 PMC4882715

[40] Dix, S. L. & Aggleton, J. P. Extending the spontaneous preference test of recognition: Evidence of object-location and object-context recognition. *Behav. Brain Res.* **99**, 191–200 (1999). 10512585 10.1016/s0166-4328(98)00079-5

[41] Yanpaisan, S. et al. Baicalin counteracts valproic acid-induced memory impairment by restoring neurogenesis in the hippocampus of adult rats. *Biomed. Pharmacother.* **192**, 118618 (2025). 41061583 10.1016/j.biopha.2025.118618

[42] Plümpe, T. et al. Variability of doublecortin-associated dendrite maturation in adult hippocampal neurogenesis is independent of the regulation of precursor cell proliferation. *BMC Neurosci* **7**, 77 (2006). 17105671 10.1186/1471-2202-7-77 PMC1657022

[43] Fuentes, R. G., Arai, M. A., Sadhu, S. K., Ahmed, F. & Ishibashi, M. Phenolic compounds from the bark of *Oroxylum indicum* activate the Ngn2 promoter. *J. Nat. Med.* **69**, 589–594 (2015). 26014045 10.1007/s11418-015-0919-3

[44] Lazarov, O. & Hollands, C. Hippocampal neurogenesis: Learning to remember. *Prog. Neurobiol.* **138–140**, 1–18 (2016). 26855369 10.1016/j.pneurobio.2015.12.006 PMC4828289

[45] Sirichoat, A. et al. Caffeic acid protects against l-methionine induced reduction in neurogenesis and cognitive impairment in a rat model. *Heliyon* **10**, e26919 (2024). 38455532 10.1016/j.heliyon.2024.e26919 PMC10918208

[46] Hsieh, H.-M., Wu, W.-M. & Hu, M.-L. Soy isoflavones attenuate oxidative stress and improve parameters related to aging and Alzheimer’s disease in C57BL/6J mice treated with D-galactose. *Food Chem. Toxicol.* **47**, 625–632 (2009). 19146912 10.1016/j.fct.2008.12.026

[47] Sreedharan, S., Pande, A., Pande, A., Majeed, M. & Cisneros-Zevallos, L. The neuroprotective effects of *Oroxylum indicum* extract in SHSY-5Y neuronal cells by upregulating BDNF Gene expression under LPS induced inflammation. *Nutrients* **16**, 1887 (2024). 38931243 10.3390/nu16121887 PMC11206423

[48] Lee, S. et al. Oroxylin A, a flavonoid, stimulates adult neurogenesis in the hippocampal dentate gyrus region of mice. *Neurochem. Res.* **35**, 1725–1732 (2010). 20680459 10.1007/s11064-010-0235-y

[49] Kim, D. H. et al. Oroxylin A enhances memory consolidation through the brain-derived neurotrophic factor in mice. *Brain Res. Bull.* **108**, 67–73 (2014). 25218897 10.1016/j.brainresbull.2014.09.001

[50] Wang, S. & Zhou, Y. Baicalein inhibits neuroapoptosis via pathways in sevoflurane induced rats. *Transl. Neurosci.* **9**, 88–98 (2018). 30042862 10.1515/tnsci-2018-0015 PMC6057263

[51] Fang, J. et al. Baicalin provides neuroprotection in traumatic brain injury mice model through Akt/Nrf2 pathway. *DDDT* **12**, 2497–2508 (2018). 30127597 10.2147/DDDT.S163951 PMC6089097

[52] Oh, S. B. et al. Baicalein attenuates impaired hippocampal neurogenesis and the neurocognitive deficits induced by γ-ray radiation. *Br. J. Pharmacol.* **168**, 421–431 (2013). 22891631 10.1111/j.1476-5381.2012.02142.x PMC3572568

[53] Gureev, A. P. et al. Age-related decline in Nrf2/ARE Signaling is associated with the mitochondrial DNA damage and cognitive impairments. *Int. J. Med. Sci.* **23**, 15197 (2022). 10.3390/ijms232315197 PMC9739464 36499517

[54] Huang, W., Zhong, Y., Gao, B., Zheng, B. & Liu, Y. Nrf2-mediated therapeutic effects of dietary flavones in different diseases. *Front. Pharmacol.* **14**, 1240433 (2023). 37767395 10.3389/fphar.2023.1240433 PMC10520786

[55] Kuwar, O. K. & Kalia, N. Anti-inflammatory and antioxidant effects of baicalein: Targeting Nrf2, and NFĸB in neurodegenerative disease. *Inflammopharmacology* **33**, 1303–1310 (2025). 40014253 10.1007/s10787-025-01698-x

[56] Liu, Z. S. J. et al. The potential of baicalin to enhance neuroprotection and mitochondrial function in a human neuronal cell model. *Mol. Psychiatry* **29**, 2487–2495 (2024). 38503930 10.1038/s41380-024-02525-5 PMC11412897

[57] Prajit, R. et al. Chrysin mitigates neuronal apoptosis and impaired hippocampal neurogenesis in male rats subjected to D-galactose-induced brain aging. *Biogerontology* **25**, 1275–1284 (2024). 39300009 10.1007/s10522-024-10140-8 PMC11486779

[58] Prajit, R. et al. Chrysin alleviates the impeded neurogenesis in accelerated brain aging by D-galactose in rats. *Biogerontology* **26**, 70 (2025). 40085327 10.1007/s10522-025-10215-0

[59] Abdulhafiz, F. et al. LC–TOF-MS/MS and GC-MS based phytochemical profiling and evaluation of wound healing activity of Oroxylum Indicum (L.) Kurz (Beka). *Front. Pharmacol.* **13**, 1050453 (2022). 36483735 10.3389/fphar.2022.1050453 PMC9723245

[60] Cheng, A. et al. Comparison of phenolic content and antioxidant capacity of red and yellow onions. *Czech J. Food Sci.* **31**, 501–508 (2013).

[61] Benzie, I. F. F. & Strain, J. J. The Ferric reducing ability of plasma (FRAP) as a measure of “Antioxidant Power”: The FRAP assay. *Anal. Biochem.* **239**, 70–76 (1996). 8660627 10.1006/abio.1996.0292

[62] Choonong, R., Jabsanthia, J., Waewaram, V., Panjanghan, K. & Putalun, W. Comparative study of callus culture and leaves of Thunbergia laurifolia for their bioactive constituents and the activation of AMPK and GLUT-dependent glucose uptake on rat skeletal muscle (L6) cells. *J Food Process Preserv* **45**, e15434 (2021).

[63] Sirichoat, A. et al. Neuroprotective properties of chrysin on decreases of cell proliferation, immature neurons and neuronal cell survival in the hippocampal dentate gyrus associated with cognition induced by methotrexate. *Neurotoxicology* **92**, 15–24 (2022). 35779630 10.1016/j.neuro.2022.06.010

[64] Antunes, M. & Biala, G. The novel object recognition memory: neurobiology, test procedure, and its modifications. *Cogn. Process.* **13**, 93–110 (2012). 22160349 10.1007/s10339-011-0430-z PMC3332351

[65] Jørn Erlandsen, E. & Randers, E. Challenges in the measurement of plasma creatinine on the Roche cobas c702. *Scand. J. Clin. Lab. Invest.* **78**, 490–495 (2018). 30261759 10.1080/00365513.2018.1501090

[66] Schumann, G. & Klauke, R. New IFCC reference procedures for the determination of catalytic activity concentrations of five enzymes in serum: preliminary upper reference limits obtained in hospitalized subjects. *Clin. Chim. Acta* **327**, 69–79 (2003). 12482620 10.1016/s0009-8981(02)00341-8

