# Correction: *Oroxylum indicum* ameliorates D-galactose-induced aging related memory impairments via enhancing rat hippocampal neurogenesis

**DOI:** 10.1038/s41598-026-36729-2

**Published:** 2026-01-20

**Authors:** Nittaya Tanrangka, Ram Prajit, Soraya Kaewngam, Tanaporn Anosri, Worapol Sae-Foo, Anusara Aranarochana, Nataya Sritawan, Apiwat Sirichoat, Waraporn Putalun, Peter Wigmore, Jariya Umka Welbat

**Affiliations:** 1 Department of Anatomy, Faculty of Medicine, Khon Kaen University, Khon Kaen, 40002 Thailand; 2 Faculty of Pharmaceutical Sciences, Khon Kaen University, Khon Kaen, 40002 Thailand; 3 Department of Pharmacognosy and Pharmaceutical Botany, Faculty of Pharmaceutical Sciences, Prince of Songkla University, Hat Yai, Songkhla Thailand; 4 School of Life Sciences, Queen’s Medical Centre, Medical School, University of Nottingham, 13, Nottingham, NG7 2RD UK

Correction to: *Scientific Reports* 10.1038/s41598-025-27042-5, published online 02 December 2025

The original version of this Article contained an error in the spelling of the author Worapol Sae-Foo, which was incorrectly given as Warapol Sae-Foo.

The original Article and accompanying Supplementary Information file have been corrected.

