# Supplementary material for: Oroxylum indicum ameliorates D-galactose-induced aging related memory impairments via enhancing rat hippocampal neurogenesis
Source: Sci Rep. 2025 Dec 2;15:43004. doi: 10.1038/s41598-025-27042-5 (PMC12673138; doi:10.1038/s41598-025-27042-5)
Supplement: Supplementary file 1 — Supplementary Material 1 [file 41598_2025_27042_MOESM1_ESM.docx]

**Supplemental material**

***Oroxylum indicum* ameliorates D-galactose-induced aging related memory impairments via enhancing rat hippocampal neurogenesis**

Nittaya Tanrangka^1^, Ram Prajit^1^, Soraya Kaewngam^1^, Tanaporn Anosri^1^, Worapol Sae-Foo^3^, Anusara Aranarochana^1^, Nataya Sritawan^1^, Apiwat Sirichoat^1^, Waraporn Putalun^2^, Peter Wigmore4, Jariya Umka Welbat1,*

^1^ Department of Anatomy, Faculty of Medicine, Khon Kaen University, Khon Kaen 40002, Thailand

^2^ Faculty of Pharmaceutical Sciences, Khon Kaen University, Khon Kaen 40002, Thailand

^3^ Department of Pharmacognosy and Pharmaceutical Botany, Faculty of Pharmaceutical Sciences, Prince of Songkla University, Hat Yai, Songkhla, Thailand

^4^ School of Life Sciences, Queen’s Medical Centre, Medical School, University of Nottingham, 13 Nottingham NG7 2RD, UK.

***Corresponding author**, e-mail: jariya@kku.ac.th; Tel.: +66-84-667-6105

**Table of content**

|  | Page |
| --- | --- |
| **Table S1** Intra- and inter-day precision of HPLC-UV system | 3 |
| **Table S2** The number of DCX-positive cells in SGZ of dentate gyrus in each group. | 4 |
| **Table S3** The number of BrdU/NeuN positive cells in SGZ of dentate gyrus in each group. | 4 |

**Table S1** Intra- and inter-day precision of HPLC-UV system

| Concentration (μg/ml) | Intra-day precision (%RSD) | | | | | Inter-day precision (%RSD) | | | | |
| --- | --- | --- | --- | --- | --- | --- | --- | --- | --- | --- |
|  | Oroxin A | Baicalin | Baicalein | Chrysin | Oroxylin A | Oroxin A | Baicalin | Baicalein | Chrysin | Oroxylin A |
| 0.75 | 1.80 | 1.61 | ND | 0.96 | 1.40 | 2.94 | 2.23 | ND | 1.41 | 0.70 |
| 6.25 | 0.79 | 0.74 | 1.00 | 0.92 | 1.78 | 2.21 | 2.63 | 0.77 | 1.21 | 0.77 |
| 12.5 | 1.34 | 0.36 | 1.64 | 0.44 | 0.45 | 0.96 | 2.96 | 0.86 | 0.89 | 1.28 |
| 25 | 0.84 | 1.29 | 0.55 | 0.12 | 0.11 | 2.10 | 1.23 | 0.75 | 2.37 | 1.37 |
| 100 | 1.58 | 1.09 | 0.65 | 1.26 | 1.20 | 1.05 | 2.88 | 2.51 | 2.55 | 2.11 |

ND: Not detection

**Table S2** The number of DCX-positive cells in SGZ of dentate gyrus in each group. All data are represented as means ± SEM.

| **Group** | **Number of DCX-positive cells** |
| --- | --- |
| 1. Vehicle | 1,009 ± 52.59 |
| 2. D-gal | 241.3 ± 47.03^****^ |
| 3. OIFE 125 | 1,182 ± 76.48 |
| 4. OIFE 250 | 1,757 ± 58.70 |
| 5. D-gal + OIFE 125 | 1,661 ± 182.2^####^ |
| 6. D-gal + OIFE 250 | 1,408 ± 50.26 ^####^ |

^####^*p<*0.0001 and **^****^** *p<*0.0001 indicates comparisons with the vehicle and D-gal groups, respectively (one-way ANOVA, Tukey’s post-hoc test).

**Table S3** The number of BrdU/NeuN positive cells in SGZ of dentate gyrus in each group. All data are represented as means ± SEM.

| **Group** | **Total number of BrdU/NeuN positive cells** |
| --- | --- |
| 1. Vehicle | 1,620 ± 128.5 |
| 2. D-gal | 868.0 ± 114.7^*^ |
| 3. OIFE 125 | 2,608 ± 148.1 |
| 4. OIFE 250 | 2,344 ± 101.2 |
| 5. D-gal + OIFE 125 | 2,311 ± 181.4^####^ |
| 6. D-gal + OIFE 250 | 2,749 ± 194.1^####^ |

^#^*p<*0.05 and ^****^ *p<*0.0001 indicates comparisons with the vehicle and D-gal groups, respectively (one-way ANOVA, Tukey’ s post-hoc test).
